# The prevalence of fibromyalgia among doctors in the tertiary care hospital: A cross-sectional study

**DOI:** 10.1016/j.amsu.2022.104931

**Published:** 2022-11-26

**Authors:** Saira Tahir, Alam Zeb, Muhammad Sharif, Obaid ur Rehman, Muhammad Sufyan khan, Mehak Mukhtar, Hassan Mumtaz, Amraha Zubair

**Affiliations:** a PGR Rheumatology PIMS, Pakistan; b Assistant Professor of Rheumatology KTH (Khyber Teaching Hospital Peshawar), Pakistan; c Registrar Rheumatology PIMS, Pakistan; d PGR Dermatology LRH, Pakistan; e Clinical Research Associate, Maroof International Hospital, Public Health Scholar: Health Services Academy, Pakistan; f Department of Internal Medicine, Dow University of Health Sciences, Pakistan

**Keywords:** Fibromyalgia, Pakistan, Widespread pain, Prevalence, Hospital

## Abstract

**Background:**

Fibromyalgia syndrome (FMS) is an umbrella term for chronic pain syndrome, associated with tenderness, fatigue, reduced pain thresholds, and paresthesia in the limbs. The field of medicine places doctors in constant work-related stress, sleep deprivation, and depression, thus increasing their vulnerability to developing FMS. This study aims to evaluate the prevalence and severity of FM (fibromyalgia) among physicians in a tertiary care hospital setting.

**Methods:**

The cross-sectional study was performed at the Pakistan Institute of Medical Sciences, Islamabad, Pakistan, from December 2019 to December 2020.243 physicians of either gender, and from all the departments being house officers, medical officers, and post-graduate trainees were included & divided into 3 age groups from 20 to more than 45 years. Widespread Pain Index (WPI) equal to or more than 7 and Symptom Severity Score (SSS) equal to or more than 5 OR WPI = 3 to 6 and SSS equal to or more than 9 were required, according to the modified American College of Rheumatology preliminary diagnostic criteria 2016 for fibromyalgia diagnosis. Data was analyzed using SPSS 25.

**Results:**

Among a total of 243 participants, FMS was diagnosed in 69 (28.40%) individuals. The predominant FMS population was the youngest age group 20–35 (56 = 81.16%). Increased BMI, increasing pain score category, gender, and comorbidities are significantly associated with FMS (p ≤ 0.05). Whereas, advancing age is insignificantly linked with FMS (p > 0.05).

**Conclusions:**

The prevalence of fibromyalgia was found to be high among doctors working in stressful hospital settings, particularly among the youngest ones.

## Highlights

• Fibromyalgia prevalence was observed in approximately one-third of doctors.
• Correct knowledge of FMS diagnostic criteria, risk factors, and treatment strategies shall be followed.

## 1 Introduction

Fibromyalgia syndrome (FMS) is a chronic medical condition that has remained a mysterious subject over the decades, with debatable etiologies [[1], [2], [3]]. The period of residency training is intensely exhausting, during which a physician learns and implements clinical knowledge [4,5]. Therefore, the hectic routine places physicians in constant sleep deprivation and psychological stress, leading to dissatisfactory work performances [4]. In addition, the psychological burden is aggravated by feelings of depression, lethargy, and burnout, followed by self-perceived major medical errors [4]. These aspects are substantially associated with FMS.

FMS is an umbrella term for centralized musculoskeletal pain disorder and encompasses mental and physical fatigue, and enhanced pain sensitivity at multiple sites, especially at tender points, mimicking neuropathic pain and paresthesia in the limbs [1,3,6]. Restless legs syndrome is reported among 30% of FMS patients [6]. Moreover, psychological perturbations including cognitive abnormalities, also known as ‘fibro fog’, sleep disturbances, like insomnia, anxiety disorders, memory impairment, and depressive tendencies are also significant manifestations [1,3,6]. Regarding abdominal and genitourinary systems, complaints of irritable bowel syndrome, dysmenorrhea and urinary urgency without urinary tract infection hold a major value [3,6].

The American College of Rheumatology, in 2010, updated the original 1990 diagnostic criteria for FMS by eliminating ‘the examination of tender point’ and instead incorporating a scoring system [3,5,7]. Although the pathophysiology of FMS is not completely perceived, the triad of central (neurologic), peripheral (inflammatory) and genetic mechanisms could be thought to play a potential role in allodynia and hyperalgesia associated with FMS [1,6]. FMS is the second most frequent disorder after osteoarthritis, leading to disability and declining quality of life [2,3,8]. FMS can be treated successfully by pharmacological, as well as non-pharmacological techniques, a point worth mentioning [3]. An estimated prevalence varies between 2% and 4% in the general population of the United States, with 15% of patients reporting in clinics [1]. Data extracted from a study involving five European countries (Italy, Germany, Spain, France, and Portugal) reported an overall frequency of 4.7% [9]. A cross-sectional study conducted in Riyadh, Saudi Arabia showed that the prevalence of FMS among physicians is 8.2%, involving primarily males [5]. In contrast, the female population is commonly affected, that is about 3-fold more than males [6]. Another similar study reported the specialty-specific prevalence of FMS in the era of stressful covid-19, demonstrating results as nursing (16.5%) and internal medicine (32.4%) [10]. However, there is limited epidemiological data involving physicians for countries in Asia. Therefore, this study focuses to assess the prevalence of fibromyalgia among physicians working in a hospital setting in Pakistan.

## 2 Methodology

This cross-sectional study was conducted at the largest federal government, tertiary care, and research-oriented hospital in Islamabad, Pakistan Institute of Medical Sciences (PIMS), from December 2019 to December 2020, catering to patients from all over the country. It includes 22 medical and surgical specialist centers, with a 592-bed facility, located in the center of Islamabad. Being Pakistan's capital and ninth-largest city, Islamabad is the home to nearly one million one hundred sixty-four thousand people. Globalization and World Cities Research Network assessed and concluded Islamabad as a Gamma + city. Being a metropolitan city, it has an urban area of 220.15 km^2^ (85.00 sq mi) and a population of 1,198,000. Moreover, 2089 inhabitants per square kilometer (5410 inhabitants per square mile) is the density of Islamabad [11].

A sample size of 243 was calculated using the Rao soft sample size calculator keeping a 95% confidence interval and 5% margin of error. Non-probability Consecutive sampling technique was used.

### 2.1 Inclusion criteria

Doctors from both genders and all the departments being house officers, medical officers, and post-graduate trainees were included in our study.

### 2.2 Exclusion criteria

The specialist consultants were excluded from our study.

### 2.3 Data collection

Data was obtained after the approval of ethical review committee wide Ref No ECPIMS/07/12, dated 2^nd^ Nov 2019 & informed consent from the individuals.

In accordance with the STROCSS 2021 recommendations [12], we conducted this study. As an added bonus, a detailed STROCSS 2021 check list may be found in the supplemental materials. UIN researchregistry8239 [13] identifies our study in Research Registry. Our research adheres to the principles outlined in the Helsinki Declaration. Ethical approval was given by KRL Hospital, Islamabad.

### 2.4 Screening techniques

The first part of the questionnaire consisted of demographic details of individuals, including age, body mass index (BMI), marital status, addictions, like smoking, and co-morbid health conditions, like hypertension, diabetes mellitus, celiac disease, inflammatory bowel disease, and syndrome (IBS and IBD), chronic medical problems, cardiac-related issues, and autoimmune disorders.

The second part enquired about the profession, financial constraints, payment status, department, place of work, whether government, private setups, or both, and family environment, whether satisfactory or not, was also asked. Furthermore, the presence or absence of pain/tenderness in many regions, including buttocks, legs, neck, back, shoulder, arm, chest, and abdomen for the past 7 days was questioned. The following symptoms were considered: fatigue, waking up tired, and having trouble thinking, with their severity assessed using the Symptom Severity Scale (SSS) indicating 0 = no problem, 1 = mild, 2 = moderate, and 3 = severe. The abdominal pain, headache, and depression incidences in the past week were too queried.

A Widespread Pain Index (WPI) score was used to evaluate the prevalence of fibromyalgia in doctors working at PIMS hospital. To fulfill the criteria, WPI equal to or more than 7 and Symptom Severity Score (SSS) equal to or more than 5 OR WPI = 3 to 6, and SSS equal to or more than 9 were needed.

Data were analyzed using SPSS version 25. Mean values were calculated & data were presented as frequencies and percentages. Chi-square test was applied among demographic and screening factors data to see the association & Odds ratio to find the risk of fibromyalgia. Pearson's correlation coefficient was employed to determine the existence of correlation. Additionally, P ≤ 0.05 was taken as statistically significant.

## 3 Results

243 (100.0%) physicians completed the questionnaire and were included in the analysis. Out of 243 (100.0%), 156 (64.20%) were males and in the majority, whereas 87 (35.8%) were females. The predominant population was the younger age group (20–35 = 75.72%), with only 7(2.88%) being aged above 45 [Table 1].

**Table 1:** Demographic details of participants.

		Number	Percentage (%)
Gender	Male	156	64.20
	FEMALE	87	35.80
Age			
Age Group	20–35	184	75.72
	36–45	52	21.40
	More Than 45	7	2.88

The overall mean BMI was 24.32 ± 6.46kg/m2, with minimum and maximum values being 14.00kg/m2 and 75.00kg/m2 respectively. The overall mean pain score was 8.26 ± 2.53, with minimum and maximum values being 1.00 to 12.00 respectively [Table 2].

**Table 2:** Further baseline details of participants.

		Number	Percentage (%)	Mean	Standard Deviation
BMI				24.32	6.46
Pain Score				8.26	2.53
	SSS≥5	69	28.40		
	SSS<5	174	71.60		
Marital Status	Married	148	60.91		
	Unmarried	89	36.63		
	Engaged	2	0.82		
	Not Specified	4	1.65		
Comorbidities	Smoking	75	30.8		

There were different Factors affecting Fibromyalgia among Doctors of PIMS, as shown in Table 3.

**Table 3:** Factors affecting Fibromyalgia among Doctors of PIMS.

		Number	Percentage (%)
Profession	Doctor	210	86.42
	Paramedical Staff	25	10.29
	Medical Student	4	1.65
	Not Specified	4	1.65
Place Of Work	Government	162	66.67
	Private	34	13.99
	Both	15	6.17
	Not Working at Present	25	10.29
	Not Specified	7	2.88
Payment Status	Paid	163	67.08
	Unpaid	70	28.81
	Not Specified	10	4.12
Financial Constraint	Yes	199	81.89
	No	42	17.28
	Not Specified	2	0.82
Family Environment	Satisfactory	217	89.30
	Unsatisfactory	22	9.05
	Not Specified	4	1.65
Department	General Medicine and Allied	135	55.56
	General Surgery and Allied	29	11.93
	Gynecology	11	4.53
	Pathology	9	3.70
	Pediatrics	15	6.17
	Emergency/Accident Emergency	4	1.65
	Prosthodontics Dentistry	1	0.41
	Medical Student/Graduate	4	1.65
	Rheumatology	3	1.23
	Medical Technology	2	0.82
	Nephrology	1	0.41
	Radiology	5	2.06
	Cardiology	3	1.23
	Administration	1	0.41
	Medical ICU	1	0.41
	Anesthesiology	1	0.41
	Orthopedics	2	0.82
	Gastroenterology	1	0.41
	Oncology	1	0.41
	Burns Center	1	0.41
	Not Working at Present	11	4.53
	Not Specified	2	0.82

The prevalence of fibromyalgia was found to be 28.40% (SSS≤5), 69 out of 243 individuals (28.40%), based on the high pain score category, meeting ACR modified 2016 criteria for FM diagnosis. The prevalence was highest among the youngest 20–35 age group (56 = 81.16%), with (7 = 10.14%) in the 35–45 age group, and the least in greater than 45 age group (1 = 1.45%). Although FMS was not significantly associated with age, the prevalence showed a decreasing trend with advancing age (p > 0.05). Moreover, it is also evidenced by the detection of the highest percentage of tenderness in various areas of the body (184 = 75.72%), and fatigue (173 = 71.20%) in the 20–35 age group. The most relevant problem reported among all participants was tenderness (243 = 100%), followed by fatigue (232 = 95.47%), waking up tired plus having trouble thinking (223 = 91.77%). Also, headache (207 = 85.19%), depression (192 = 79.01%), and abdominal cramps (191 = 78.60%) were highlighted. The increased frequency of physicians (58 = 23.87%) reported pain in the lower legs, while the least percentage reported pain in the buttocks (14 = 5.76%). Among all age groups, many (102 = 41.98%) reported severe fatigue non-significantly (p = 0.204). Further, FMS symptoms, such as having trouble thinking, waking up tired, pain in the abdomen, and depression were significantly linked to age (p ≤ 0.05), whereas headache and co-morbid conditions, like smoking (75 = 30.86%) and rheumatoid arthritis (168 = 69.14%) showed an insignificant link with age (p > 0.05). Moreover, the increased tenderness frequencies were significantly observed in males (156 = 64.20%, p = 0.026). FMS severity symptoms: fatigue, trouble thinking, waking up tired, and pain in the abdomen were significantly correlated with gender, while depression, headache, and co-morbidities showed an insignificant association. The majority (213 = 87.65%) reported pain of high intensity (pain score = 6–10), while few (30 = 12.35%) reported pain of low intensity (pain score = 1–5), thus pain score category showed a statistically significant relationship with tenderness (p = 0.026). The pain score category was also significantly linked to the above-mentioned symptoms associated with the severity of FMS (p ≤ 0.05) [Table 4].

**Table 4:** Association of Demographics with the Screening Factors (*) Significant results, p ≤ 0.05.

Screening Factors Used	Gender			Age Groups				Pain Score Category			BMI Category		
	Male	Female	P Value	20–35	36–45	Above 45	P Value	1–5	6–10	P Value	Less Than 25	More Than 25	P Value
Tenderness In Body	156	87	0.026*	184	52	7	0.160	30	213	0.026*	127	116	0.030*
Fatigue	145	87	0.016*	173	52	7	0.204	22	210	0.000*	116	116	0.002*
Trouble Thinking	138	85	0.003*	164	52	7	0.032*	21	202	0.000*	109	114	0.000*
Waking Up Tired	140	83	0.018*	164	52	7	0.002*	17	206	0.000*	111	112	0.000*
Cramps In Abdomen	104	87	0.000*	132	52	7	0.000*	1	190	0.000*	75	116	0.000*
Depression	123	69	0.072	141	46	5	0.049*	16	176	0.000*	96	96	0.002*
Headache	129	78	0.391	150	49	6	0.369	18	189	0.000*	101	106	0.039*
Comorbidities	156	87	0.160	184	52	7	0.728	30	213	0.001*	127	116	0.107

The most relevant problem reported among fibromyalgia diagnosed individuals is waking up tired (69 = 100%), followed by having trouble thinking (66 = 95.65%), fatigue (63 = 91.3%), tenderness (53 = 76.81%), headache (52 = 75.36%), depression (31 = 44.93), and lastly abdominal pain (23 = 33.33%). Thus, the severity symptoms demonstrated a significant association with FMS (p ≤ 0.05).

Logistic regression analysis demonstrated that gender (p = 0.160 OR = 1.52, CI = 0.85–2.73), and body mass index (BMI) (p = 0.107 OR = 1.57, CI = 0.91–2.73) categories, when compared with co-morbidities, were not associated with greater odds of being positive for FM. Whereas comparing pain scores (p = 0.001 OR = 3.50, CI = 1.60–7.64), we found increased odds. Comparing BMIs, many (127 = 52.26%) were healthy (BMI less than 25), while some (116 = 47.74%) had BMI greater than 25. BMI category was statistically significant to the presence of FMS; tenderness, fatigue, trouble thinking, waking up tired, abdominal cramps, headache, and depression (p ≤ 0.05), but not with co-morbidities (p > 0.05) [Table 5].

**Table 5:** Prevalence of Fibromyalgia (FM) based on Symptom Severity Score (SSS).

	FM Diagnosed		FM not Diagnosed	
	Number	Percentages (%)	Number	Percentages (%)
	69	28.40	174	71.60
**Tenderness**	53	76.81	16	23.19
**Fatigue**	63	91.30	6	8.70
**Waking up Tired**	69	100.00	0	0.00
**Trouble Thinking**	66	95.65	3	7.69
**Abdominal Pain**	23	33.33	46	66.67
**Headache**	52	75.36	17	24.64
**Depression**	31	44.93	38	55.07

## 4 Discussion

The objective of this cross-sectional study was to assess the prevalence of fibromyalgia among doctors working in hectic hospital schedules. As per our awareness, this is the first-ever study investigating the prevalence of fibromyalgia among doctors in a healthcare setting in Pakistan. This population is especially susceptible to chronic significant stress, impaired life quality, fatigue, sleep issues, decreased pain thresholds, and oversaturation, negatively impacting their professional and personal lives and endeavors [5].

The prevalence reported in our study was 28.40%. This is lower than another study done among visiting patients conducted at PIMS hospital (33%) and another study performed by Javed and colleagues (55.80%) [1,14]. Moreover, the prevalence in our research was greater than that found in the general population (4.4%) [15]. However, opposite to our results, several studies have previously reported frequencies like the general population. In one such study comprising 306 medical students in Turkey, the frequency was observed to be 2% [16]. The variations between our study outcomes and that of Eyigor et al. could potentially be explained by differences between ACR 1990 and ACR modified 2016 criteria [16].

An extensive literature regarding pathophysiological mechanisms of FMS has been documented, although the exact mechanisms remain unknown. Awareness of underlying mechanisms paves a clear path toward the appropriate management goals. Evidence from the biochemical and imaging studies suggests the phenomenon of ‘central nervous system sensitization’ plays an important role, which describes the abnormalities of ascending and descending tracts [1,3]. This leads to augmented response to mechanical stimulation, hyperalgesia with decreased pain thresholds, allodynia, and autonomic nervous system abnormalities [1,3].

Moreover, alterations in the peripheral nervous system, neuroendocrine activation, and hyperfunctioning of cells of innate and adaptive immunity, such as dendritic cells, mast cells, and T lymphocytes respectively, are observed [3]. Likewise, this is accompanied by the release of an enormous deal of neuropeptides, like glutamate, neurokinin A, brain-derived neurotrophic factor (BDNF), and substance P, favoring the ‘neuroinflammation’, which is triggered by multiple noxious stimuli, both environmental and psychological [3]. Our study found that ‘waking up tired’ was the most common symptom among the fibromyalgia diagnosed group. This is supported by the potential role of substance P in causing disturbed sleep, in a comparative study done by Anderson et al. [17] Further, Moldofsky et al. showed that FMS manifestations, including increased tenderness, muscle pain, and decreased pain thresholds are strongly correlated with sleep disturbance [18]. Also, Papp et al., using the Epworth Sleepiness Scale (ESS), assessed the sleeping pattern of resident physicians and surprisingly found that approximately 84% of physicians demonstrated deranged scores, indicating the causal roles of sleep deprivation and fatigue in adversely affecting physicians' lives [19].

The possibility that fibromyalgia has a strong genetic predisposition is evidenced by a study conducted by Arnold et al., demonstrating an elevated (13.6%) fibromyalgia risk. among siblings [20]. This can be attributed to specific genes, particularly linked with the physiology of neurotransmitters [1]. Although females are shown to be more prone to developing FM, the predominant population in our study consisted of males. Also, fibromyalgia was relatively more frequent among the youngest population (20–30), as opposed to the usual age of presentation in the general population [15]. Increased prevalence in males can be explained by the constant stress of bread-earning responsibility for their families, and certainly a communication gap between healthcare providers and FM-diagnosed males [21]. Our study mimics a study conducted by Omair et al., showing the high FM prevalence among Saudi Arabian residents in training [5]. Opposite to our findings, Patel et al. found the raised FM percentages among females and the older population [22].

Another noticeable aspect of our study is the significant association between increasing BMI and FMS. The leading factors favoring obesity in doctors, including male gender, family history of obesity, consuming snacks between meals, and poor physical activity are worth mentioning [23]. Congruent to our study, another study demonstrated the high risk of FMS, bad mood, and worse functional outcomes associated with increased BMI, and thus emphasized its treatment [24]. Besides, the biochemical markers, such as apolipoprotein B, and CRP are also related to FMS-related dysfunction in normal and obese individuals, thus indicating significantly higher odds of cardiovascular events [25].

Our study demonstrated a statistically significant link between FMS and comorbidities. The probable mechanisms explain that tobacco smoking affects cognitive function as due to extreme pain sensitization, it consumes the neurotransmitters, thus decreasing the neural resources needed for cognitive function [26]. Nicotine is indicated in causing derangements of neurotransmitters, including dopamine, serotonin, and noradrenaline, leading to defective pain modulation [27].

The latest medical literature suggests that FM-related inflammation is caused by plasma-derived factors cytokines, reactive oxygen species, and lipid mediators, thus opening doors to effective therapeutic approaches [28]. Also, antioxidants, as treatment options are encouraged, as FMS leads to disbalances between malondialdehyde, an oxidant, and superoxide dismutase, an antioxidant [29].

Our study has certain limitations, including involving a single center, less sample population, and the study design being cross-sectional, leading to the inability to detect the exact causal association among the analyzed aspects.

## 5 Conclusions

FM prevalence was observed in approximately one-third of doctors working in a tertiary care hospital. Thereby, categorizing this population as the most vulnerable FM group. The correct management requires comprehensive knowledge of FMS diagnostic criteria, risk factors, and treatment strategies. Further research is encouraged to shed light on FMS etiology, pathophysiology, new treatments, preventive approaches, and associated aggravating factors, like oxidative stress, smoking, and other addictions.

## Ethical approval

Data was obtained after the approval of ethical review committee wide Ref No ECPIMS/07/12, dated 2^nd^ Nov 2019 & informed consent from the individuals.

## Sources of funding

Nill.

## Author contribution

Contributorship Statement:

Substantial contributions to the conception or design of the work; or the acquisition, analysis, or interpretation of data for the work: Saira Tahir.

Drafting the work or revising it critically for important intellectual content: Alam Zeb, Muhammad Sharif, Obaid ur Rehman.

Final approval of the version to be published: Hassan Mumtaz, Muhammad Sufyan khan.

Agreement to be accountable for all aspects of the work in ensuring that questions related to the accuracy or integrity of any part of the work are appropriately investigated and resolved: Mehak Mukhtar, Amraha Zubair.

## Registration of research studies

1. Name of the registry: Research registry
2. Unique Identifying number or registration ID: researchregistry8239Browse the Registry - Research Registry.

## Guarantor

Muhammad Sharif, Alamzeb.

## Consent

The informed consent from the patients was obtained considering Helsinki's Declaration.

## Provenance and peer review

Not commissioned, externally peer-reviewed.

## Availability of data and materials

Data sharing does not apply to this article as no datasets were generated or analyzed for the current report.

## Declaration of competing interest

Nill.
